# Antifungal Activity of Bioactive Metabolites Produced by *Trichoderma* *asperellum* and *Trichoderma* *atroviride* in Liquid Medium

**DOI:** 10.3390/jof6040263

**Published:** 2020-11-01

**Authors:** Claudia Stracquadanio, Juan Manuel Quiles, Giuseppe Meca, Santa Olga Cacciola

**Affiliations:** 1 Department of Agricultural Science, Mediterranean University of Reggio Calabria, Localitá Feo di Vito, 89122 Reggio Calabria, Italy; claudia.stracquadanio@unirc.it; 2 Department of Agriculture, Food and Environment, University of Catania, Via S. Sofia 100, 95123 Catania, Italy; 3 Department of Preventive Medicine, University of Valencia, Av. Vicent Andrés Estellés s/n, 46100 Burjassot, Valencia, Spain; juan.quiles@uv.es (J.M.Q.); giuseppe.meca@uv.es (G.M.)

**Keywords:** *Trichoderma asperellum*, *Trichoderma atroviride*, bioactive metabolites, biological control, plant pathogens

## Abstract

*Trichoderma* spp. are known as biocontrol agents of fungal plant pathogens and have been recognized as a potential source of bioactive metabolites. The production of antimicrobial substances from strains *T*. *atroviride* (TS) and *T*. *asperellum* (IMI 393899) was investigated. The bioactivity of 10- and 30-day culture filtrate extracted with ethyl acetate was assessed against a set of pathogenic fungi and oomycetes. The 30-day extracts of both strains had significant cytotoxic effects against the tested pathogens, with values of minimum fungicidal concentration (MFC) ranging between 0.19 and 6.25 mg/mL. Dual culture assay (direct contact and nondirect contact) and the percentage inhibition of radial growth (PIRG) was calculated. The highest PIRG values were 76% and 81% (direct contact) with IMI 393899 and TS, respectively. Nondirect contact does not show inhibition on any of pathogens tested, indicating that the inhibition is not due to the secretion of volatile substances. Culture filtrates were analyzed by GC-MS and HPLC-Q-TOF-MS for the identification of volatile organic compounds (VOCs) and nonvolatile organic compounds (nVOCs), respectively. Seven classes of VOCs and 12 molecules of nVOCs were identified. These results indicate that these strains of *Trichoderma* had antimicrobial activities and they are potential natural sources of compounds with biological activity.

## 1. Introduction

Synthetic chemicals, such as fertilizers, herbicides and pesticides, used in horticultural production systems and during postharvest processes are environmental pollutants and potentially harmful to humans and animals [1]. The excessive use of broad-spectrum synthetic fungicides can affect the microbiome of the rhizosphere, including symbiotic fungi and beneficial bacteria. These microorganisms interact with the plants, modifying and promoting the availability of nutrients in the soil, such as nitrogen fixation [2]. In addition, numerous studies have shown that there is the risk of selection of resistant strains of pathogens to synthetic fungicides [3]. The excessive use of synthetic chemicals in farming systems has led governmental and international institutions to limit their use and shift interest towards sustainable food and agriculture (FAO, 2020). A valid alternative to synthetic fungicides is the use of biocontrol agents and/or their metabolites. Currently, several biocontrol agents are recognized including bacterial, such as *Agrobacterium*, *Bacillus,* and *Pseudomonas,* and fungal, such as *Ampelomyces*, *Aspergillus*, *Candida*, *Coniothyrium*, *Gliocadium*, *Pseudozyma*, *Streptomyces,* and *Trichoderma,* agents [4]. In particular, the *Trichoderma* genus have ability to adapt and thrive in different environmental conditions. This genus proved effective in the sustainable management of crop diseases caused by fungi. *Trichoderma* spp. are among the most frequently isolated soil inhabiting fungi and are common in the plant rhizosphere. These fungi are opportunistic, avirulent plant symbionts, and act as parasites and antagonists of many phytopathogenic fungi, thus protecting plants from disease [5]. The antagonistic properties of *Trichoderma* spp. are based on the activation of indirect and direct mechanisms. The indirect mechanisms are competition for space and nutrients, promotion of growth, and induction of plant defenses, whereas the direct mechanisms are mycoparasitism and production of active metabolites and lytic enzymes. These indirect and direct mechanisms can act synergistically and depending on species and strain [6]. An interesting feature of *Trichoderma* spp. is the production of volatile and nonvolatile secondary metabolites capable of inhibiting the growth of pathogens. However, little is known about their production which can vary from strain to strain. Based on their structure, known metabolites with antibiotic activity can be classified into two main types: (i.) low-molecular-weight and volatile metabolites and (ii.) high-molecular-weight and polar metabolites. Low molecular weight and volatile metabolites include simple aromatic compounds, volatile terpenes, and isocyanide, and relatively nonpolar substances with significant vapor pressure [7]. These ‘‘volatile organic compounds’’ in the soil environment would be expected to diffuse over a distance through systems thus enhancing the performance of the organism by affecting the physiology of competitor organisms [7,8]. High-molecular-weight and polar metabolites, like peptaibols, may exert their activity through direct interactions between *Trichoderma* species and their antagonists [7]. In this study, we tested the antagonistic ability and the production of secondary metabolites with inhibitory activity of strains of *Trichoderma asperellum* and *Trichoderma atroviride* on different pathogens of the genera *Fusarium*, *Aspergillus*, *Penicillium*, *Colletotrichum*, *Neofusicoccum,* and *Phytophthora*, that cause loss to agricultural crops.

## 2. Materials and Methods

### 2.1. Chemical Materials

HPLC-grade methanol and acetonitrile and analytical reagent grade ethyl acetate, formic acid (99%), and dimethyl sulfoxide (99.9% DMSO) were obtained from Thermo Fisher Scientific (Loughborough, UK). Magnesium sulfate (MgSO_4_) was obtained from Thermo Fisher Scientific (Kandel, Germany). Potato dextrose agar (PDA) and Potato dextrose broth (PDB) was obtained from Thermo Fisher Scientific (Basingstoke, UK). Ultrapure water (<18 MΩ/cm) was obtained from a Milli-Q purification system (Millipore Corp., Bedford, MA, USA).

### 2.2. Fungal Strains, Culture Conditions, and Spore Production

Six *Penicillium* strains (*P. digitatum* CECT 2954, *P. commune* CECT 20767, *P. expansum* CECT 2278, *P. roqueforti* CECT 2905, *P. camemberti* CECT 2267, *P. brevicopactum* CECT 2316) and four *Aspergillus* strains (*A. parasiticus* CECT 2681, *A. niger* CECT 2088, *A. steynii* CECT 20510, *A. lacticoffeatus* CECT 20581) were sourced from the Spanish Type Culture Collection (Valencia, Spain).

Six Fusarium strains (*F. proliferatum* ITEM 12072, F. verticillioides ITEM 12052, *F. sporotrichoides* ITEM 12168, *F. langsethiae* ITEM 11031, *F. poae* ITEM 9151, *F. graminearum* ITEM 126) and two Aspergillus strains (*A. flavus* ITEM 8111, *A. carbonarius* ITEM 5010) were obtained from the Agro-Food Microbial Culture Collection (Bari, Italy).

*Penicillium verrucosum* VTT D-01847 was obtained from the VTT Culture Collection (Finland). Two *Neofusicoccum* species (*N. batangarum*, *N. parvum*), two *Colletotrichum* species (*C. acutatum*, *C. gloeosporioides*), two species of the oomycete *Phytophthora* (*Ph. parvispora*, *Ph. nicotianae*) and two species of *Trichoderma* (*T. asperellum* IMI 393899, *T. atroviride* TS) were obtained from the collection of the Molecular Plant Pathology Laboratory of the Di3A, University of Catania (Catania, Italy).

The mycotoxigenic fungi were cryopreserved in sterile 30% glycerol at –80 °C, but before antifungal studies they were defrosted and cultured in PDB at 25 °C for 48 h and inoculated on PDA plates to obtain spores.

All other fungal strains were maintained on potato dextrose agar (PDA) at room temperature and subcultures were made every 20 days.

The strains of *Neofusicoccum* and *Colletotrichum* were grown on oatmeal agar (OMA) to increase the production of conidia [9].

*Phytophthora nicotianae* and *Ph*. *parvispora* were grown on OMA for the production of sporangia. From 10-day-old cultures, a zoospore suspension was obtained by flooding each culture plate with 10 mL sterile distilled water. The plates with the sterile distilled water were then refrigerated (5 °C) for 25 min and incubated in the dark at 25 °C for 30 min to induce zoospore release [10].

The OMA was prepared with the protocol [11] slightly modified. In particular, 60 g of rolled oats were added to 600 mL of water and then heated for 40 min. The suspension was sieved and 12 g of agar was added, subsequently reaching the final volume of 1 L and autoclaved for 20 min at 121 °C. The OMA medium for *Phytophthora* was prepared as previously described, but with 75 g of rolled oats and 20 g of agar [11].

### 2.3. Isolation and Identification of Trichoderma Strains

The strain of *Trichoderma asperellum,* IMI 393899 [12,13], had previously been identified morphologically as *Trichoderma harzianum*. However, recent DNA analysis has identified it as *T*. *asperellum*. *Trichoderma atroviride,* TS strain, was isolated from the basidiocarp of *Ganoderma lucidum* a wood parasite. The tissue fragments of the basidiocarp (5 mm), taken between the healthy tissue and the infected tissue, were washed with 1% NaClO for 2 min, rinsed with sterile distilled water and transferred onto plates with potato dextrose agar (PDA) with the addition of streptomycin sulfate (0.25 g/L). The plates were incubated at 25 °C for 24 h. Pure subcultures were obtained from growing colonies on PDA.

For the identification of *Trichoderma* spp. the growing mycelium on PDA plates was taken and DNA was extracted and purified using the DNA PowerPlant^®^ Pro Kit (Qiagen, Milan, Italy). The identification of *Trichoderma* isolates was carried out by amplification and analysis of the regions of the Internal Transcribed Spacer (ITS) region of ribosomal DNA (rDNA). The amplification was made using Taq DNA polymerase, recombinant (Invitrogen™, Milan, Italy) with the universal primer pairs ITS-5 (5′-GGAAGTAAAAGTCGTAACAAGG-3′) and ITS-4 (5′-TCCTCCGCTTATTGATATGC-3′) [14].

The reaction mixture for PCR amplification was PCR buffer (1X), dNTP mixture (0.2 mM), MgCl_2_ (1.5 mM), forward and reverse primers (0.5 mM), Taq DNA Polymerase (1 U), and 100 ng of DNA.

The conditions of the thermocycler were: 94 °C for 3 min; followed by 35 cycles of 94 °C for 30 s, 55 °C for 30 s and 72 °C for 30 s, and finally, 72 °C for 10 min. The amplicons obtained were confirmed in 1% agarose gel and sequenced in both directions by an external service (Macrogen, Amsterdam, the Netherlands). Obtained sequences were analyzed using FinchTV v.1.4.0 [15]. For species identification, blast searches in GenBank [16] were performed.

### 2.4. Liquid Culture and Metabolite Production

Two plugs of each *Trichoderma* strains, obtained from actively growing margins of PDA cultures, were used to inoculate 750 mL flasks containing 500 mL of sterile potato dextrose broth (PDB). The liquid cultures were incubated for 10 and 30 days at 30 °C under stirring (100 rpm). The cultures were filtered under vacuum through filter paper, and the filtrates were stored at 2 °C for 24 h before the biphasic extraction. Then 10- and 30-day culture filtrates (100 mL) and the relative PDB (controls) were stored at −80 °C and, subsequently, freeze-dried for the analysis of volatile organic compounds (VOCs).

### 2.5. Extraction of Metabolites from Liquid Culture

The 10- and 30- day culture filtrates of *T*. *asperellum* IMI 393899 and *T*. *atroviride* TS were extracted with ethyl acetate (EtOAc) for 3 times with a final 1:1 ratio.

The combined organic fraction was dried (MgSO_4_) and evaporated under reduced pressure at 35 °C. The red-brown residues recovered were dissolved with 10% DMSO or MeOH and stored at -20 °C until the subsequent analysis was done.

### 2.6. Agar Diffusion Test

The activity of EtOAc extracts of 10 and 30 days of the two *Trichoderma* strains was tested against different pathogens with the agar diffusion test. The spore suspension of pathogen was stratified on the surface of PDA plate using sterile cotton swabs and 10 µL of extract at different concentrations (0, 1, 10, 25, 50, and 100 mg/mL) were distributed on the surface. The plates were incubated at 25 °C. The control 10% DMSO was used in the test. After 72 h of incubation, the diameter of the inhibition halos was measured. *Phytophthora* species are oomycetes, which form zoospores as infectious propagules and have a different behavior from spores. For this reason, the assay for two species of *Phytophthora* was performed in a different way. A plug (5 mm) of mycelium from an active growing culture was placed in the center of the PDA plate, and 10 µL of extract was placed at a distance of 25 mm using the concentrations previously reported. The plates were incubated at 25 °C for 3 days. The inhibition of the growth was assessed by measuring the diameter of the inhibition halos in comparison with the control (10% DMSO).

### 2.7. Antimicrobic Dilution Assay

The minimum inhibitory concentration (MIC), defined as the lowest concentration of the treatment that inhibits the visible fungal growth, was determined with a microdilution method. A 100 μL aliquot of 30-day EtOAc extract (stock solution: 50 mg/mL), diluted with sterile PDB to obtain final concentrations of 0.09–25 mg/mL, was added to 96-well sterile microplates. Then, the wells were inoculated with 100 μL of a 5 × 10^4^ spores (or zoospores)/mL suspension and incubated at 25 °C for 72 h. The suspension of the pathogen strains was prepared by suspending the spores or zoospores in buffered peptone water with 1% Tween 20. Spore or zoospores were counted using the Neubauer chamber and the suspension was adjusted to the final concentration [17]. Wells with either PDB and the spores (or zoospores) of the pathogens or PDB and 10% DMSO were used as controls.

After determining the MIC, aliquots of the wells with concentrations corresponding to the MIC as well as with higher concentrations were used to inoculate PDA plates for the determination of the minimum fungicidal concentration (MFC). After incubation of the plates at 25 °C for 72 h, the MFC, defined as the lowest treatment concentration required to kill a pathogen and corresponding to a nonvisible growth of the subculture, was determined. Three replicates of each assay were assessed.

### 2.8. Dual Culture Assay

The competition ability for direct contact and production of nonvolatile or volatile substances by *Trichoderma* strains were evaluated with two methods of dual culture assay.

In both methods, a 5 mm plug, taken from 5 to 7 days cultures on PDA of the pathogen as well as of the antagonist, was placed on the opposite side of a PDA plate; however, in the second method, the plate was separated into two compartments. The first method evaluates the inhibition growth for direct contact and production of nonvolatile substances, whereas the second method determines the possibility of inhibition due to volatile substances, because they are not in direct contact.

All plates were incubated at 25 °C and radial growth of pathogens was measured daily. The controls were plates with only the pathogen and three repetitions were made per thesis.

The percentage inhibitory of radial growth (PIRG) was calculated using the following formula:

$$\mathrm{PIRG}\%\;=\;\frac{\left(\mathrm{Dc}-\mathrm{Dt}\right)}{\left(\mathrm{Dc}\right)}\times 100,$$

where PIRG is percent of growth inhibition;

Dc is growth rate of the pathogen (control);

Dt is growth rate of the pathogen in presence of *Trichoderma.*

### 2.9. Analysis of VOCs

Lyophilized culture filtrate (200 mg) was mixed with 2 mL of water and placed in a 10 mL glass vial. VOCs were identified by gas chromatography with a single quadrupole mass spectrometer detector (GC/MS) analysis. Prior to analysis, samples were incubated in a water bath at 55 °C for 45 min, while being gently stirred with a rod. VOCs were extracted from the vial headspace by solid-phase microextraction (SPME). An SPME holder (Supelco, Bellefonte, PA, USA) containing a fused-silica fiber coated with a 50/30 μm layer of divinylbenzene/Carboxen/polydimethylsiloxane (DVB/CAR/PDMS) was used to trap VOCs in the vial headspace. The fiber was introduced into the splitless inlet of an Agilent 6890N GC system (Agilent Technologies, Palo Alto, Santa Clara, CA, USA), and thermal desorption of the analytes was performed at 250 °C for 5 min. The GC system was equipped with an HP-5MS (30 m × 0.25 mm and 0.25 μm 5% diphenyl/95% dimethylpolysiloxane) capillary column (J&W Scientific, Folsom, CA, USA). The oven was programmed to start at 40 °C (held for 2 min) and to ramp up to 160 °C at 6 °C/min, and then, it was increased to 260 °C at 10 °C/min (held for 4 min). Helium (99.999%) was used as the carrier gas, and the flow rate was 1 mL/min. The flow was transferred from the column into an Agilent 5973 MS detector (Agilent Technologies, Palo Alto). The ion source temperature was set at 230 °C, the ionizing electron energy was 70 eV, and the mass range was 40–450 Da in full scan acquisition mode. Compounds were identified using the NIST Atomic Spectra Database version 1.6 (Gaithersburg, MD, USA), considering spectra with 95% similarity. Results were expressed as a percentage of the VOC by dividing the area of each peak by the total area of the chromatogram peaks [18]. The analysis was carried out in triplicate. 

### 2.10. Analysis of nVOCs

For the identification of nonvolatile organic compounds (nVOCs), 50 mg of the crude extracts were resuspended with 2 mL of methanol. All samples were filtered through 0.22 μm nylon membrane prior to injection. The HPLC system used for the chromatographic determination was an Agilent 1200 (Agilent Technologies, Santa Clara, CA, USA) equipped with a vacuum degasser, autosampler, and binary pump. The column was a Gemini C18 (50 mm × 2 mm, 100 Å, and 3 μm particle size; Phenomenex, Torrance, CA, USA).

The binary mobile phases consisted of water (A) and acetonitrile (B) with 0.1% v/v formic acid. The initial gradient of the mobile phase was 5% B for 5 min and was increased to 95% B over 10 min. It was maintained at 95% B for 20 min, reduced to 5% B for 5 min, and then maintained for 5 min. The flow rate was maintained at 0.3 mL/min and 20 μL of each sample was injected.

Mass spectrometry (MS) analysis was performed using a Q-TOF-MS (6540 Agilent Ultra High Definition Accurate Mass, Santa Clara, CA, USA), equipped with an Agilent Dual Jet Stream electrospray ionization (Dual AJS ESI, Santa Clara, CA, USA) interface in positive and negative ion mode over the range of m/z 50–1500. The parameters were as follows: drying gas flow (N2), 8.0 L/min; nebulizer pressure, 30 psig; gas drying temperature, 350 °C; capillary voltage, 3.5 kV; and fragmentor voltage, 175 V. Targeted MS/MS experiments were carried out using collision energy values of 10, 20, and 40 eV. Integration and data elaboration were managed using MassHunter Qualitative Analysis software B.08.00 (Agilent, Santa Clara, CA, USA) [19]. The analysis was carried out in triplicate.

## 3. Results and Discussion

### 3.1. Antifungal Activity on Solid Medium, MIC, and MFC

The results of the antifungal activity of nonvolatile or diffusible compounds obtained with the two *Trichoderma* strains tested, *T. asperellum* IMI 393899 and *T. atroviride* TS, showed that the extracted antifungal compounds inhibited the growth of all pathogens in the agar diffusion assay, as shown in Table 1.

The agar diffusion test of the extract of the 10 days culture filtrate at maximum concentrations incited a change in the colony morphology of the pathogen, but not a significant reduction in growth. For this reason, all subsequent inhibition tests were carried out using 30-day extracts. Both 30-day EtOAc extracts were effective on all tested pathogens, showing inhibition halos that varied with the concentration of the extract.

In particular, the extract of *T*. *asperellum* showed an inhibition halo at a concentration of 1 mg/mL on *P*. *expansum*, *P*. *camemberti*, *A*. *niger*, *A*. *steynii,* and *A*. *carbonarius*, with an average halo of 8 mm. The inhibition was visible on all pathogens at a concentration of 10 mg/mL with an average halo of 9.7 mm. At this concentration, the most susceptible was *A*. *steynii* with an area of inhibition of 17 mm and the least susceptible was *C*. *gloesporioides* with an area of inhibition of 5 mm. At the maximum concentration of 100 mg/mL extract, the average inhibition values were 24 mm and exceeded 30 mm for of *P*. *camemberti*, *A*. *niger*, *A*. *steynii*, *Neofusicoccum* spp., *Ph*. *Parvispora,* and *Ph. nicotianae*. At this concentration, the lowest value was 18 mm of inhibition area for *P*. *verrucosum* and *F*. *poae*.

The extract of *T*. *atroviride* was effective at the minimum concentration of 10 mg/mL on all pathogens with the exception of *A*. *parasiticus*, *F*. *verticillioides*, *F*. *langsethiae,* and *F*. *graminearum*; moreover, *A*. *parasiticus* was not inhibited at the concentration of 25 mg/mL and the inhibition halo was observed at concentrations of 50 and 100 mg/mL with values of 7 and 11 mm, respectively.

At a concentration of 10 mg/mL, the average inhibition area of all pathogens was 7 mm, the lowest value was 4 mm for *F*. *proliferatum* and the highest value was 13 mm for *Ph*. *nicotianae*. For the maximum tested concentration of 100 mg/mL, the mean inhibition area was 14 mm, species of *Neofusicoccum* were the most susceptible to the extracts with an inhibition halo of 27 mm, while the minimum value of 10 mm was obtained on *P*. *brevicopactum*, *F*. *langsethiae,* and *F*. *poae*.

Both extracts were shown to have inhibitory activity on all tested pathogens. In particular, the extract of *T*. *asperellum* was more effective, as it was able to inhibit at the minimum concentration (10 mg/mL) of *A*. *parasiticus*, the major fungus responsible for aflatoxin formation in crop seeds [20].

**Figure 1:**
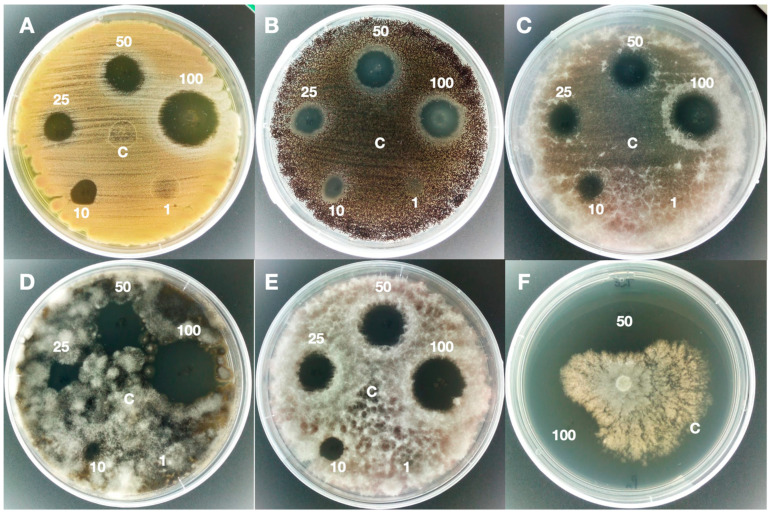
Agar diffusion test of *Trichoderma asperellum* IMI393899 extract. The picture shows the halos of antifungal activity at different concentrations (1, 10, 25, 50, and 100 mg/mL) of 30-days EtOAc extract and in comparison with the "C" control (10% DMSO) on *Penicillium camemberti* (**A**), *Aspergillus carbonarius* (**B**), *Fusarium verticillioides* (**C**), *Neofusicoccum parvum* (**D**), *Colletotrichum acutatum* (**E**), and *Phytophthora nicotianae* (**F**).

**Figure 2:**
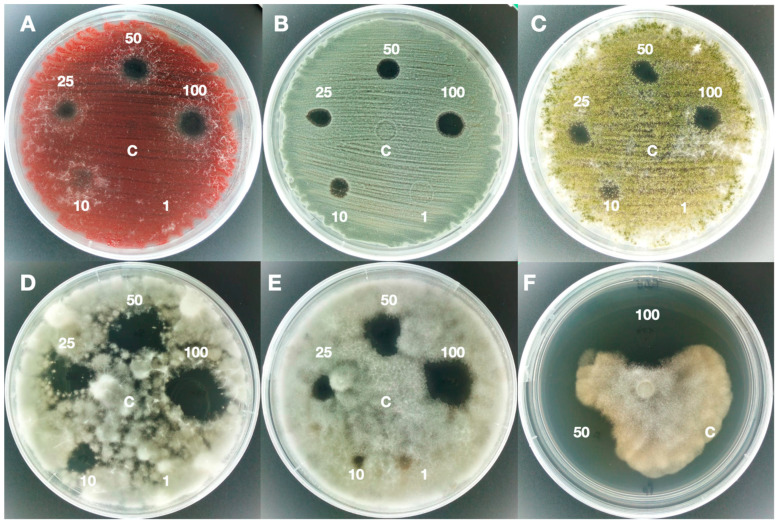
Agar diffusion test of *Trichoderma atroviride* TS extract. The picture shows the halos of antifungal activity at different concentrations (1, 10, 25, 50, and 100 mg/mL) of 30-days EtOAc extract and in comparison with the "C" control (10% DMSO) on *Fusarium proliferatum* (**A**), *Penicillium brevicopactum* (**B**), *Aspergillus Flavus* (**C**), *Neofusicoccum batangarum* (**D**), *Colletotrichum gloeosporioides* (**E**), and *Phytophthora parvispora* (**F**).

Interestingly, a week after test initiation, the inhibition halo persisted (Figure 1 and Figure 2), thus showing that these extracts have a great inhibition potential in vitro.

The results of the quantitative analysis of the EtOAc extracts obtained with the MIC and MFC are shown in Table 2. The MIC of *T*. *asperellum* IMI 393899 extract ranged between 0.09 and 0.78 mg/mL and values of MFC were in the range of 0.19–1.56 mg/mL.

The extract of *T*. *atroviride* TS, despite higher concentrations, proved effective with values in the range of 0.19–3.13 and 0.39–6.25 mg/mL of MIC and MFC, respectively.

*Penicillium* spp. were the most susceptible to the antifungal activity of both extracts; these pathogens are among the most common in postharvest rot, which cause significant economic damage due to loss of production [21].

### 3.2. Antagonistic Properties

In the first method, inhibition by direct contact and/or by secretion of metabolites, both *Trichoderma* species showed an inhibition of the mycelial growth of all the tested pathogens. Table 3 shows the PIRG observed on the first, third, and seventh day of the test. In some cases, the inhibition is observed from the first day and, as matter of fact, it appears that the growth rate of the pathogen could influence the efficiency of *Trichoderma* in inhibiting the growth of the pathogen. This observation is supported by Vinale et al. [22] who demonstrated that the production and the class of metabolites produced by *Trichoderma* are influenced by the presence and type of host pathogen.

In the case of *Penicillium* strains with a slower growth rate, the inhibition is observed only on the third day when *Trichoderma* has filled half of the plate and is close to the pathogen.

On the seventh day, the highest PIRG was observed when *Trichoderma* came into direct contact with the pathogen. In particular, both *Trichoderma* strains showed PIRG values higher than 40% and the maximum values of PIRG were obtained for *Ph*. *nicotianae* with 76% and 81% for *T*. *asperellum* and *T*. *atroviride*, respectively. Only *P*. *expansum* showed less than 20% radial growth inhibition. 

**Figure 3:**
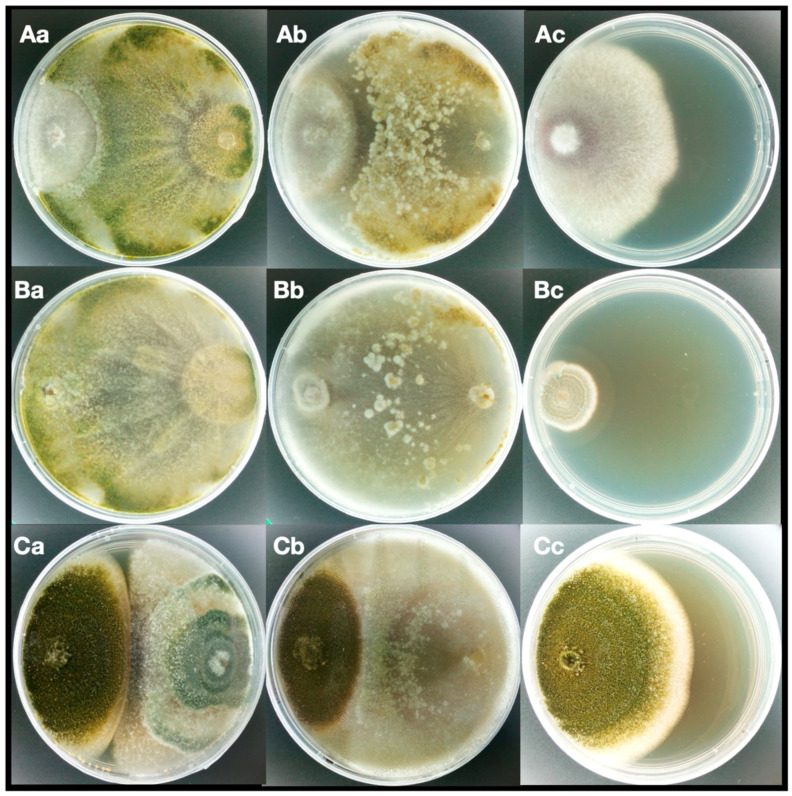
Dual culture assay after 7-days of incubation. *Fusarium graminearum* (**A**), *Penicillium commune* (**B**), and *Aspergillus parasiticus* (**C**) in coculture (left side of each plate) with *Trichoderma atroviride* TS (**a**), *Trichoderma asperellum* IMI393899, (**b**) and the control (**c**).

In Figure 3, a 7-day test of *F*. *graminearum*, *P*. *commune,* and *A*. *parasiticus* grown with *T*. *atroviride* and *T*. *asperellum* clearly demonstrates the inhibition of the growth in comparison with the control (only the pathogen). Control cultures of the tested pathogens showed a faster growth with respect to dual culture. The dual culture plates showed rapid initial growth of the fungus that stopped at the point of contact with the antagonist. In some cases, the biological antagonist was not only able to inhibit the mycelium growth of the pathogen but also it grew on it, causing degradation of the mycelium (Table 3), as in the case of *P*. *commune* (Figure 3Ba).

According to the generally accepted definition, antibiosis is the mechanism mediated by specific metabolites such as enzymes, volatile compounds, and nonvolatile antibiotics [23]. The involvement of enzymes in biological control complicates the distinction between mycoparasitism and antibiosis.

Mycoparasitism is one of the main strategies of *Trichoderma* spp. to antagonize pathogens as supported by previous studies. John et al., 2010 [24] reported mycoparasitic activity from *Trichoderma viride* on *Pythium arrhenomanes* and *Fusarium oxysporum* f. sp. *adzuki*; *Trichoderma* showed a mycoparasitic activity on both pathogens, but mainly on *Pythium*. Krauss et al., 1998 [25], showed that the MP11 variety of *Trichoderma* spp. was able to mycoparasitize various pathogens (*Fusarium* spp., *Colletotrichum* spp., *Botryodiplodia theobromae,* and *Nigrospora sphaerica*). In another study, Hibar et al., 2005 [26], showed the invasion of the mycelium of *Trichoderma harzianum* on *Fusarium oxysporum* f. sp. *radicis*-*lycopersici* cultures after 6 days in direct contact. Microscopic observations, made at the level of the contact area between *T*. *harzianum* and *F*. *oxysporum* f. sp. *radicis*-*lycopersici*, showed a profound change in the mycelium of the pathogen characterized by significant lysis, in which the hyphae of *Trichoderma* were enveloping those of the pathogen. In the study of Tondje et al., 2007 [27], in which the four strains of *Trichoderma* were used to control the black pod disease of cocoa caused by a *Phytophthora megakarya*, the biological principle of their actions was discussed. According to these authors, the mycoparasitic activity of the *T*. *asperellum* strains involves both direct penetration and enveloping the hyphae of the pathogen; in the latter case, a high level of hydrolytic enzymes is produced before the destruction of the hyphae.

In the second method of the double culture test, which makes it possible to evaluate the inhibition by secretion of volatile substances, no inhibition was observed for the 3 days in which the test was conducted (data not shown). This may suppose either that the inhibition of the two strains of *Trichoderma, T. asperellum* IMI 393899 and *T. atroviride* TS, is not mainly due to the secretion of volatile substances or that antibiotic volatile substances are not normally produced if *Trichoderma* is not approaching the pathogen. 

Calistru et al., 1997 [28], showed that the inhibition growth of *A*. *flavus* and *F*. *moniliforme* was due to volatile substances produced by two strains of *T*. *harzianum* (T1 and T2) and two strains of *T*. *viride* (T5 and T6). The growth of *A*. *flavus* and *F*. *moniliforme* was inhibited when the colonies were exposed to the trapped atmosphere from cultures of *Trichoderma* spp., with the exception of *T*. *harzianum* T1. *Trichoderma harzianum* T2 only inhibited the growth of *A*. *flavus*, while *T*. *harzianum* T1 did not reduce the growth of *F*. *moniliforme* and *A*. *flavus*. This shows that not all *Trichoderma* strains are able to inhibit the in vitro growth by producing volatile substances.

### 3.3. Identification of VOCs and nVOCs

A total of 62 VOCs was identified in the lyophilized samples of *Trichoderma* strains and only seven were also present in the control medium (PDB). The VOCs identified in the lyophilized culture filtrates of the two *Trichoderma* strains and the control medium with a percentage > 0.01% are shown in Table 4. The identified compounds are classified according to the chemical class into alcohols, aldehydes, acids, ketones, pyrazines, esters, and others.

Both culture filtrates presented high levels of alcohols and pyrazines compared to the control. Among the compounds most identified in *T*. *asperellum* IMI 393899 culture filtrate were 3-methyl-1-butanol (4.47%), phenylethyl alcohol (19.06%), tetramethyl pyrazine (4.81%), 6-pentyl-alpha-pyrone (42.66%), α-Zingiberene (5.86%), and α-Sesquiphellandrene (6.60%), while in the culture filtrate of *T*. *atroviride* TS were 3-methylbutanol (5.29%), tetramethylpyrazine (56.79%), and 1-Hydroxy-2.4-di-tert-butylbenzene (6.17%).

The compound 6-pentyl-alpha-pyrone (6-PP) is an unsaturated lactone with a strong coconut-like aroma, which was first characterized by Collins & Halim (1972) [29], and identified to be one of the bioactive compounds of several species of *Trichoderma*, such as *T*. *viride* [29], *T*. *harzianum* [30,31], and *T*. *atroviride* [32,33]. The biological effects of 6-PP are the reduction of the production of the mycotoxin deoxynivalenol by *F*. *graminearum* [34] and the fusaric acid by *F*. *moniliform* [35]; moreover, it exerts antifungal properties, reducing the mycelial growth rate of *Rhizoctonia solani* and *F*. *oxysporum* f. sp. *lycopersici* [36]. Finally, Vinale et al., 2008 [37] reported that 6-PP has a growth-promoting effect on tomato seedlings which compared to control plants had a more extensive and vigorous root system.

Tetramethylpyrazine, also known as Lanzorite, is a compound belonging to the alkylpyrazines with the characteristic nutty and roasted flavor [38]; it has been found in fermented cocoa beans and soybeans fermented by *Bacillus* spp. [39,40]. There is no report in the literature on this substance produced by *Trichoderma* spp.

Pyrazines are known as substances with antifungal and nematicidal activity [41,42], previous studies have shown that tetramethylpyrazine inhibits the growth of *Moniliophthora perniciosa* and *F*. *oxysporum* f. sp. *lactucae* [43].

The analysis of the nonvolatile organic compounds (nVOCs) of the culture filtrates of *Trichoderma* strains tested, *T. asperellum* IMI 393899 and *T. atroviride* TS, led to the identification of 12 molecules present in the literature, namely, diterpene lactone trichodermaerin, peptabolite asperilins A, H, and E, dicheto pyrazine methylcordysinin A, steroids 3,5,9-trihydroxyergosta-7,22-dien-6-one and ergosta-7,22-dien-3-ol, sterol beta-sitosterol, adenine nucleoside, cyclopentenone atrichodermone B, sesquiterpene atrichodermone C, and the pyrone derivative 6-penta-1-enyl-pyran-2-one.

**Figure 4:**
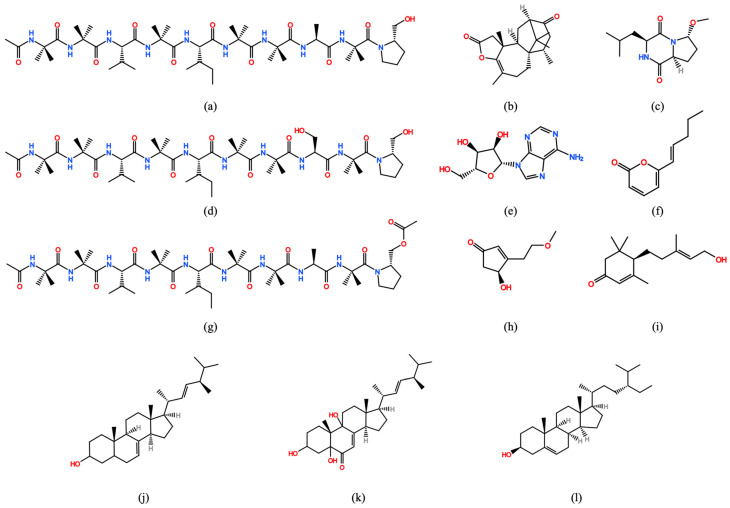
Chemical structure of the nonvolatile compounds (nVOCs) identified in the *Trichoderma asperellum* (IMI 393899) and *Trichoderma atroviride* (TS) EtOAc extracts. (**a**) aspereline A; (**b**) trichodermaerin; (**c**) methylcordysinin a; (**d**) aspereline E; (**e**) adenine nucleoside; (**f**) 6-pent-1-enyl-pyran-2-one; (**g**) aspereline H; (**h**) atrichodermone B; (**i**) atrichodermone C; (**j**) ergosta-7,22-dien-3-ol; (**k**) 3,5,9-trihydroxyergosta-7,22-dien-6-one; and (**l**) beta-sitosterol.

The molecules identified in the extract after 10 and 30 days and their molecular structure are shown in Table 5 and in Figure 4. All the molecules known for their antimicrobial activity were recovered in the extract of the culture filtrate after 30 days with the exception of beta-sitosterol and ergosta-7,22-dien-3-ol found in the 10-day extracts. This could explain why the 30-day extracts were more active than the 10-day ones. In particular, 3,5,9-trihydroxyergosta-7,22-dien-6-one was isolated from *Trichoderma* spp. by Xuan et al., 2014 [44] and showed antimicrobial properties against *E*. *coli*, *B*. *subtilis*, *P*. *oryzae*, *C*. *albicans*, *A*. *niger,* and *A*. *alternata*. Beta-sitosterol isolated from *T*. *asperellum* [45] and *T*. *harzianum* [46] showed inhibitory activity against *R*. *solani*, *S*. *rolfsii*, *M*. *phaseolina,* and *F*. *oxysporum* [46].

However, a few studies on the identification of nVOCs produced by *Trichoderma* spp. are present in the literature and many more studies should be performed to understand the antimicrobial activity of the secondary metabolites produced.

## 4. Conclusions

The two *Trichoderma* strains tested in this study, *T. asperellum* IMI 393899 and *T. atroviride* TS, inhibited the growth of tested pathogens when they came in contact with them. In particular, the extract of *T*. *asperellum* showed the highest inhibition activity and was active even at a low concentration. Moreover, it was demonstrated that both *Trichoderma* strains produced compounds with antifungal activity against the pathogenic fungi and oomycetes tested. 

These results highlight the potential use of these two *Trichoderma* strains as antagonists in biological control of plant pathogens and their ability to produce secondary bioactive metabolites that might be used for the management of crop and postharvest diseases as an alternative to synthetic fungicides.

## Figures and Tables

**Table 1:** Agar diffusion test of 30-days EtOAc extracts. Several concentrations of the EtOAc extracts of *Trichoderma asperellum* IMI393899 and *T*. *atroviride* TS were tested on different pathogens; results were measured as the diameter of the inhibition halos. * Range below 5 mm (-), 5–10 mm (+), range 11–15 mm (++), range 16–20 mm (+++), range 21–25 mm (++++), and more than 26 mm (+++++).

Strains	* EtOAc Extracts of											
	*T. asperellum*							*T. atroviride*				
Concentrations mg/mL	0	1	10	25	50	100	0	1	10	25	50	100
*Penicillium digitatum*	-	-	+	++	+++	+++	-	-	+	++	++	++
*Penicillium commune*	-	-	+	+++	+++	+++	-	-	+	+	++	++
*Penicillium expansum*	-	+	++	++	+++	+++	-	-	+	++	++	++
*Penicillium roqueforti*	-	-	+	++	++	++++	-	-	+	+	+	++
*Penicillium camemberti*	-	++	++	+++	++++	+++++	-	-	+	+	++	++
*Penicillium brevicopactum*	-	-	+	++	+++	++++	-	-	+	+	+	++
*Penicillium verrucosum*	-	-	++	++	++	+++	-	-	+	+	+	++
*Aspergillus parasiticus*	-	-	+	+	+++	++++	-	-	-	-	+	++
*Aspergillus flavus*	-	-	++	++	++	++++	-	-	+	+	++	++
*Aspergillus niger*	-	+	+	++++	++++	+++++	-	-	+	+	++	++
*Aspergillus steynii*	-	++	+++	+++++	+++++	+++++	-	-	+	++	++	++
*Aspergillus carbonarius*	-	+	+	+++	+++++	++++	-	-	+	+	+	++
*Aspergillus lacticoffeatus*	-	-	++	+++	++++	+++++	-	-	+	+	++	++
*Fusarium proliferatum*	-	-	++	++	+++	+++	-	-	+	+	++	++
*Fusarium verticillioides*	-	-	+	++	+++	++++	-	-	-	+	++	++
*Fusarium sporotrichoides*	-	-	++	++	+++	++++	-	-	+	+	+	++
*Fusarium langsethiae*	-	-	+	+	++	+++	-	-	-	+	++	++
*Fusarium poae*	-	-	++	++	++	+++	-	-	+	+	++	++
*Fusarium graminearum*	-	-	+	++	+++	++++	-	-	-	+	++	++
*Neofusicoccum batangarum*	-	-	+	+++	+++++	+++++	-	-	+	+++	++++	+++++
*Neofusicoccum parvum*	-	-	+	+++	+++++	+++++	-	-	+	+++	++++	+++++
*Colletotrichum acutatum*	-	-	+	++	+++	+++	-	-	+	+	++	+++
*Colletotrichum gloeosporioides*	-	-	+	+	+++	+++++	-	-	-	+	++	+++
*Phytophthora parvispora*	-	-	++	+++	++++	+++++	-	-	+	++	++	++++
*Phytophthora nicotianae*	-	-	++	+++	++++	+++++	-	-	++	++++	+++	+++++

**Table 2:** Minimum inhibitory concentration (MIC) and minimum fungicidal concentration (MFC). Values of the EtOAc extracts of *Trichoderma asperellum* IMI393899 and *T. atroviride* TS on tested fungus and oomycete pathogens.

Strains	EtOAc Extract of			
	*T. asperellum*		*T. atroviride*	
	MIC	MFC	MIC	MFC
*Neofusicoccum batangarum*	0.39	0.78	3.13	3.13
*Neofusicoccum parvum*	0.78	0.78	3.13	6.25
*Colletotrichum acutatum*	0.39	1.56	1.56	3.13
*Colletotrichum gloeosporioides*	0.39	1.56	1.56	3.13
*Phytophthora parvispora*	0.39	0.78	1.56	3.13
*Phytophthora nicotianae*	0.19	0.78	1.56	3.13
*Penicillium digitatum*	0.09	0.39	0.19	0.39
*Penicillium commune*	0.09	0.39	0.19	0.39
*Penicillium expansum*	0.09	0.39	0.19	0.78
*Penicillium roqueforti*	0.09	0.39	0.19	0.39
*Penicillium camemberti*	0.19	0.78	0.39	0.78
*Penicillium brevicopactum*	0.09	0.39	0.19	0.78
*Penicillium verrucosum*	0.39	0.78	1.56	1.56
*Aspergillus parasiticus*	0.39	0.78	3.13	6.25
*Aspergillus flavus*	0.39	0.78	3,13	6.25
*Aspergillus niger*	0.39	0.78	1.56	3.13
*Aspergillus steynii*	0.09	0.19	0.78	1.56
*Aspergillus carbonarius*	0.39	0.78	1.56	3.13
*Aspergillus lacticoffeatus*	0.39	0.78	3.13	3.13
*Fusarium proliferatum*	0.39	0.78	0.78	0.78
*Fusarium verticillioides*	0.78	0.78	0.78	1.56
*Fusarium sporotrichoides*	0.19	0.78	0.19	0.78
*Fusarium langsethiae*	0.39	1.56	0.78	1.56
*Fusarium poae*	0.39	0.78	0.78	0.78
*Fusarium graminearum*	0.39	0.78	0.78	1.56

**Table 3:** The percentage inhibition of radial growth (PIRG) in dual culture assay. Results are expressed as the ± standard error of PIRG after 1-, 3-, and 7-day incubation; * Represents pathogens that showed signs of mycelial lysis due to mycoparasitism by *Trichoderma asperellum* IMI393899 and *T. atroviride* TS; ^a^ Not Determined

Strains	*Trichoderma asperellum*			*Trichoderma atroviride*		
	First Day	Third Day	Seventh Day	First Day	Third Day	Seventh Day
*Neofusicoccum batangarum*	3.64 ± 1.82	37.78 ± 2.00	63.75 ± 2.6 *	14.55 ± 3.64	36.67 ± 1.67	67.50 ± 1.44 *
*Neofusicoccum parvum*	6.45 ± 3.23	43.75 ± 0.83	54.17 ± 0.42 *	8.06 ± 2.79	41.35 ± 0.48	63.33 ± 2.32 *
*Colletotrichum acutatum*	ND ^a^	3.17 ± 1.59	46.28 ± 2.19	ND	ND	47.93 ± 1.43
*Colletotrichum gloeosporioides*	ND	10.26 ± 1.28	59.01 ± 0 *	13.33 ± 3.33	14.10 ± 1.28	58.39 ± 2.24
*Phytophthora parvispora*	ND	9.09 ± 0	48.62 ± 0.92 *	ND	9.09 ± 0	44.95 ± 0
*Phytophthora nicotianae*	5.77 ± 1.92	22.39 ± 1.49	76.24 ± 2.97 *	7.69 ± 0	25.37 ± 1.49	81.19 ± 0.99 *
*Penicillium digitatum*	ND	3.03 ± 1.52	56.52 ± 1.26 *	ND	ND	71.01 ± 2.61 *
*Penicillium commune*	ND	20.00 ± 4.00	51.85 ± 1.85	ND	12.00 ± 4.00	68.52 ± 1.85 *
*Penicillium expansum*	ND	ND	18.18 ± 3.15	ND	ND	30.91 ± 7.93
*Penicillium roqueforti*	ND	ND	51.67 ± 0.83 *	ND	7.02 ± 1.75	75.83 ± 6.01 *
*Penicillium camemberti*	ND	ND	25.71 ± 12.45	ND	9.09 ± 4.55	28.57 ± 10.30
*Penicillium brevicopactum*	ND	11.11 ± 5.56	43.90 ± 2.44	ND	22.22 ± 14.70	68.29 ± 4.88
*Penicillium verrucosum*	ND	15.79 ± 5.26	42.42 ± 6.06 *	ND	21.05 ± 9.12	63.64 ± 5.25 *
*Aspergillus parasiticus*	2.70 ± 0	4.82 ± 1.20	46.05 ± 0.66	13.51 ± 2.70	16.87 ± 7.23	50.00 ± 7.59
*Aspergillus flavus*	ND	4.11 ± 1.37	38.52 ± 0	ND	4.11 ± 2.74	22.95 ± 3.57
*Aspergillus niger*	ND	8.99 ± 1.95	45.86 ± 1.69	ND	ND	41.40 ± 3.87
*Aspergillus steynii*	ND	ND	42.22 ± 1.11	ND	ND	25.56 ± 1.11 *
*Aspergillus carbonarius*	ND	6.82 ± 1.14	40.58 ± 0.72	ND	ND	23.91 ± 2.17
*Aspergillus lacticoffeatus*	ND	4.11 ± 2.74	34.55 ± 1.57	8.11 ± 2.70	9.59 ± 4.11	21.82 ± 0.91
*Fusarium proliferatum*	ND	10.94 ± 2.71	50 ± 0.96 *	ND	6.25 ± 0	42.31 ± 1.67 *
*Fusarium verticillioides*	7.69 ± 0	9.59 ± 0	45.83 ± 3.00	7.69 ± 0	8.22 ± 1.37	35.83 ± 1.67 *
*Fusarium sporotrichoides*	ND	5.88 ± 3.11	50 ± 2.47 *	ND	3.53 ± 1.18	39.73 ± 1.37 *
*Fusarium langsethiae*	ND	1.67 ± 1.67	42.57 ± 0.99 *	ND	3.33 ± 1.67	30.69 ± 5.51
*Fusarium poae*	ND	12.82 ± 5.13	67.15 ± 2.53 *	ND	2.56 ± 1.28	48.91 ± 1.93 *
*Fusarium graminearum*	ND	3.03 ± 3.03	61.19 ± 5.22 *	ND	6.06 ± 1.52	51.49 ± 1.97

**Table 4:** Volatile organic compounds (VOCs) produced in the culture filtrate of *Trichoderma asperellum* IMI393899 and *T*. *atroviride* TS identified by GC-MS. Results are expressed as mean relative abundance percentages (as obtained by dividing the area of each peak by the total area of the chromatogram peaks) ± standard deviation.

Compound (VOCs)	Strains	
	*Trichoderma asperellum*	*Trichoderma atroviride*
*Alcohols*		
Ethanol	0.58 ± 0.01	1.02 ± 0.07
2-methyl-1-propanol	0.70 ± 0.01	0.68 ± 0.05
3-methyl-1-butanol	4.47 ± 0.13	5.29 ± 0.23
3-methyl acetate-1-butanol	0.35 ± 0.01	ND
2-methyl acetate-1-butanol	0.18 ± 0.01	ND
Benzyl alcohol	0.35 ± 0.05	ND
Phenylethyl Alcohol	19.06 ± 0.08	ND
1-phenyl-2-propanol	ND *	0.15 ± 0
2-(4-methoxyphenyl)ethanol	0.71 ± 0.03	ND
*Aldehydes*		
Benzaldehyde	0.13 ± 0	0.20 ± 0
Benzeneacetaldehyde	0.06 ± 0	0.91 ± 0.04
3-methyl-benzaldehyde	0.30 ± 0.01	ND
Nonanal	ND	0.11 ± 0
*Acids*		
1-methyl-6-oxopyridine-3-carboxylic acid	ND	0.20 ± 0.01
*Ketones*		
2,3-butandione	ND	1.25 ± 0.04
3-ethyl-2-cyclopenten-1-one	0.41 ± 0.03	0.87 ± 0.08
Phenylacetone	ND	0.23 ± 0.02
5,6,6-trimethyl-3,4-undecadiene-2,10-dione	0.27 ± 0.02	1.55 ± 0.02
*Pyrazines*		
Trimethyl pyrazine	0.09 ± 0.01	0.43 ± 0.03
Tetramethyl pyrazine	4.81 ± 0.04	56.79 ± 0.29
2,3,5-trimethyl-6-ethylpyrazine	0.45 ± 0.01	1 ± 0.06
2,3,5-trimethyl-6-propylpyrazine	0.14 ± 0	0.14 ± 0.01
2-methyl-3,5-diethylpyrazine	ND	0.74 ± 0.02
Trimethylisobutylpyrazine	ND	0.86 ± 0.07
2,3-dimethyl-5-(1-propenyl)pyrazine	0.04 ± 0	ND
*Esters*		
Ethyl acetate	0.62 ± 0.01	0.46 ± 0
Ethyl isobutyrate	0.15 ± 0.02	ND
Isobutyl butanoate	0.22 ± 0.01	ND
Ethyl β-hydroxybutyrate	0.11 ± 0.01	0.22 ± 0
Isoamyl 2-methylpropanoate	0.18 ± 0.01	ND
Butyl isobutyrate	0.10 ± 0	ND
Ethyl 3-acetoxybutanoate	ND	0.34 ± 0.01
β-phenylethyl formate	0.14 ± 0.01	ND
Ethyl benzeneacetate	ND	1.16 ± 0.09
2-phenethyl acetate	1.15 ± 0.05	ND
Pentanoic acid, oct-4-yl ester	ND	0.66 ± 0.07
Isobutyl phenylacetate	ND	0.08 ± 0.01
α-phenylethyl butyrate	0.41 ± 0.01	ND
Butyl adipate	ND	0.26 ± 0.02
*Others*		
2,2-diphenyl-2H-1-benzopyran	0.22 ± 0.02	ND
Trimethyloxazole	ND	0.83 ± 0.03
2-pentyl furan	0.19 ± 0.01	ND
Methyl hydroxytriazaindolizine	0.08 ± 0	ND
5,6,7,8-tetrahydro-2-methyl-4H-chromen-4-one	0.23 ± 0.01	1.38 ± 0.08
Methyl 3,4-di-O-acetyl-2-O-methylfucofuranoside	0.16 ± 0.01	ND
1,3-di-tert-butylbenzene	0.20 ± 0.02	0.48 ± 0.18
2,4-dimethyl-3-acetylpyrrole	ND	0.29 ± 0.04
6,7-dimethyl-1,2,3,5,8,8a-hexahydronaphthalene	0.22 ± 0.03	ND
3-methylhexyl isothiocyanate	ND	1.39 ± 0.10
6-pentyl-alpha-pyrone	42.66 ± 0.15	0.26 ± 0
α-Himachalene	2.14 ± 0.13	0.61 ± 0.01
α-Zingiberene	5.86 ± 0.01	2.67 ± 0.14
1-hydroxy-2,4-di-tert-butylbenzene	2.50 ± 0.04	6.17 ± 0.04
Butylated hydroxytoluene	0.58 ± 0.01	1.66 ± 0.11
α-Sesquiphellandrene	6.60 ± 0.07	2.43 ± 0.05
Nerolidol	ND	0.86 ± 0.01
Cedrene	0.34 ± 0.08	ND
6,7-dihydro-2-trans-farnesol	0.63 ± 0.04	ND
1,2,4,4,6-pentamethyl-1,4-dihydropyridine-3,5-dicarbonitrile	ND	1.19 ± 0.01
Ledol	0.95 ± 0.02	3.04 ± 0.36
Phenol, 2-(1,1-dimethylethyl)-4-(1-methyl-1-phenylethyl	0.26 ± 0	1.15 ± 0.76

* Not Determined.

**Table 5:** Nonvolatile compounds (nVOCs) in the EtOAc extracts after 10 and 30 days of incubation identified by HPLC-Q-TOF-MS.

Compound (nVOCs)	MW	Molecular Formula	m/z	Ion	RT	Days of Fermentation	Reference
Trichodermaerin	316.20	C_20_ H_28_ O_3_	317.2104	[M+H]^+^	15.9	30	[47]
Aspereline H	978.62	C_47_ H_82_ N_10_ O_12_	977.6099	[M-H]^−^	16.7	10	[48]
Aspereline A	936.60	C_45_ H_80_ N_10_ O_11_	935.5956	[M-H]^−^	19.3	10	[48]
Aspereline E	952.60	C_45_ H_80_ N_10_ O_12_	951.5935	[M-H]^−^	16.6	10	[48]
Methylcordysinin A	240.15	C_12_ H_20_ N_2_ O_3_	299.1628	[M+CH3COO]^−^	20.7	10	[49]
3,5,9-trihydroxyergosta-7,22-dien- 6-one	444.32	C_28_ H_44_ O_4_	489.3224	[M-HCOO]^−^	9.4	30	[44]
Ergosta-7,22-dien-3-ol	398.36	C_28_ H_46_ O	397.3486	[M-H]^−^	30.3	10–30	[45]
Beta-sitosterol	414.39	C_29_ H_50_ O	413.3784	[M-H]^−^	33.3	10	[47]
Adenine nucleoside	267.10	C_10_ H_13_ N_5_ O_4_	312.0953	[M-HCOO]^−^	5.5	30	[45]
Atrichodermone B	156.08	C_8_ H_12_ O_3_	215.0914	[M+CH3COO]^−^	9.3	30	[50]
Atrichodermone C	236.18	C_15_ H_24_ O_2_	295.1906	[M+CH3COO]^−^	23.5	30	[50]
6-pent-1-enyl-pyran-2-one	164.08	C_10_ H_12_ O_2_	165.0915	[M+H]^+^	9.9	30	[51]
